# Natural Revegetation Alters Habitat Conditions, Bacterial Components, and Polycyclic Aromatic Hydrocarbon (PAH)-Degrading Communities in Aged PAH-Polluted Soils

**DOI:** 10.3390/microorganisms13051098

**Published:** 2025-05-09

**Authors:** Jinrong Huang, Heng Liang, Lilong Huang, Qi Li, Lei Ji, Yingna Xing, Chang Zhou, Jianing Wang, Xiaowen Fu

**Affiliations:** 1 Shandong Province Key Laboratory of Applied Microbiology, Ecology Institute, Qilu University of Technology (Shandong Academy of Sciences), Jinan 250103, China; 10431221175@stu.qlu.edu.cn (J.H.); liqi@qlu.edu.cn (Q.L.); jilei.1010@163.com (L.J.); ouertingliu@163.com (Y.X.); 13053399704@163.com (C.Z.); wangjn@sdas.org (J.W.); 2 Shandong Environmental Sciences Environmental Engineering Co., Ltd., Jinan 250109, China; sdhkylh@126.com; 3 School of Environmental Science and Engineering, Shandong University, Qingdao 266237, China; sduhll@126.com

**Keywords:** aged contamination, PAH-degrading bacteria, vegetation, saline soil

## Abstract

The vegetation restoration of contaminated sites plays a critical role in ensuring the sustained stability and functional integrity of natural ecosystems. However, during the natural revegetation process, the variations in habitat conditions, bacterial community structure, and metabolic functions in aged, polluted soil are still unclear. In the present study, we investigated aged, polycyclic aromatic hydrocarbon (PAH)-polluted soils at closed, abandoned oil well sites from the Yellow River Delta. Using gene amplification and real-time qPCR methods, the abundance, taxonomy, and diversity characteristics of indigenous bacterial communities and functional bacteria carrying C12O genes in both vegetated soils and bare soils were investigated. The results show that natural revegetation significantly changes the physicochemical parameters, PAH content, and bacterial community structure of aged, PAH-polluted soils. When comparing the abundance and components of PAH-degrading bacterial communities in vegetated and bare soils, the PAH-degrading potential was revealed to be stimulated by vegetation communities. Through correlation analysis, dual stress from soil salinity and PAH contamination in bacterial communities was revealed to be mediated through alterations in the soil’s physicochemical properties by local vegetation. The network analysis revealed that bacterial communities in vegetated soils have higher network connectivity. These results elucidate the alterations in habitat conditions, bacterial components, and PAH-degrading communities following vegetation restoration, providing critical insights for optimizing ecological rehabilitation strategies in salinized and contaminated ecosystems.

## 1. Introduction

Polycyclic aromatic hydrocarbons (PAHs) have been classified as typical persistent organic pollutants (POPs) because of their widely recognized characteristics of toxicity, mutagenicity, carcinogenicity, and recalcitrance [1,2]. In oilfields, PAHs are the most concerning natural components in crude oil and usually emerge in soils because of improper operations during petroleum exploitation and petrochemical production [3]. Following the decommissioning of oil wells with subeconomic reserves, PAH contamination originating from drilling operations is observed to enter a phase of natural aging and progressive degradation. Through ecological succession, vegetation communities undergo gradual restoration, ultimately evolving into a mosaic landscape characterized by alternating bare and vegetated patches [4,5].

The natural environment possesses the inherent ability to assimilate and manage a certain level of PAH contamination [6]. Through exposure to exogenic substances in the soil, soil bacteria can immediately shift their community and structure. Previous research has demonstrated that native microorganisms carrying PAH-degrading genes are capable of utilizing these hydrocarbons as energy sources via heterotrophic metabolism or co-metabolic processes [7]. Therefore, the transformation of microbial communities is, in essence, the transfer of energy towards the metabolic processes of microorganisms that carry relevant genes [8]. Based on the primary metabolic pathways of PAHs, the PAH-RHD and C12O genes that encode ring-hydroxylation dioxygenases and the catechol 1, 2-dioxygenase enzymes, respectively, are widely recognized as critical factors governing PAH biodegradation [9,10]. By analyzing the microbial communities carrying these genes and their abundance in soil, it is possible to elucidate the natural environment’s adaptive mechanisms in response to exogenous pollutant exposure.

In nature, plants and microorganisms form a symbiotic ecosystem through a series of complex interactions [11]. On the one hand, vegetation provides a suitable environment for functional microorganisms in the rhizosphere and strongly influences their composition in the soil [12]. On the other hand, microorganisms promote plant growth by providing bioavailable nutrients through their metabolic processes, including nitrogen, phosphorus, organic matter, and so on [13,14]. Previous studies have begun to explore this synergistic symbiotic relationship between plants and microorganisms, and to co-apply them in the remediation of organic pollutants in soil [15,16]. Wang et al. [17] revealed that PAHs entering soil are able to clearly affect vegetation and microbial communities in the soil environment. Li et al. [5] pointed out that the concentration of PAHs in vegetated soil is significantly lower than that in non-vegetated soil. However, the mechanisms by which natural restored plants and microorganisms exert their respective functions and interact to counteract the negative effects of soil pollutants and abiotic stress remain largely unknown [18].

The Yellow River Delta (YRD) of China is a typical coastal oilfield region, scattered with numerous abandoned oil well sites that prevent the natural aging of contaminants and restoration of the ecosystem. Due to the unique geological features of the YRD, soil ecosystems in these sites are subjected to dual stress from both PAH contaminants and salt-induced pressures. Elucidating the impact of revegetation on bacterial community composition, structure, and functionality in naturally aged soils would provide critical insights into the interactions between vegetation and microbial consortia during natural succession in soil matrices. In the present study, the characteristics of PAHs and bacterial communities in aged PAH-contaminated soils in the Shengli Oilfield in the YRD were investigated. By comparing the abundance and community structure of PAH-degrading bacteria carrying PAH-RHDα and C12O genes in different conditions of salinity, the influence of vegetation on the indigenous functional bacteria was explored. The results could help in understanding the natural behavior of microbial communities in response to environmental stresses and benefit the management and bioremediation of PAH contamination in soils with adverse environments.

## 2. Materials and Methods

### 2.1. Study Area and Sampling

**Figure 1:**
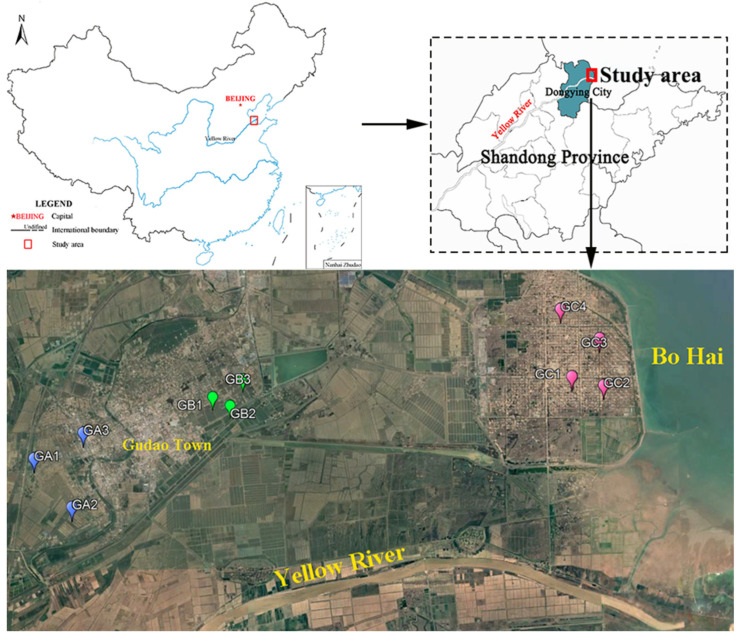
Sampling sites of aged PAH-polluted soils in the Shengli Oilfield.

PAH-contaminated soils from decommissioned well sites of the Shengli Oilfield in the YRD were sampled to investigate aging characteristics under a natural revegetation process. On 29 September 2023, we selected 20 sampling sites and collected 40 soil samples (0~20 cm depth) from both vegetated and bare land at closed, abandoned wells in this area (Figure 1, Table S1). Two samples of bare soil and two samples of vegetated soil were collected, respectively, at 10 sites. One sample was used for the determination of soil properties and the other for the determination of microbial communities. As the distance between the sampling site and coastline decreased, the sampling regions were sequentially termed GA, GB, and GC; from these sites, 3 groups of soil samples with a gradient of soil salinity were obtained, while samples of bare soil were assigned as GAB, GBB, and GCB and samples of vegetated soil were coded as GAV, GBV, and GCV. The vegetation community in the vegetated soil of the sampling area was predominantly composed of Phragmites australis, Suaeda heteroptera, and Tamarix chinensis. Three sub-samples were collected at each sampling point as parallel samples using a stainless-steel shovel and then uniformly mixed with equal amounts. All samples were collected away from obvious tar blocks and crude oil spillage. During the transport process to the laboratory, soil samples were stored in sterilized plastic tubes at −20 °C. In the laboratory, each sample was divided into two portions. One portion was freeze-dried and finely ground (passing through a 2 mm sieve) for determinations of PAHs and physiochemical properties, and the other was stored at −80 °C, preparing for DNA extraction and molecular microbiological analysis.

### 2.2. Analysis of Physicochemical Properties and PAHs

The physicochemical properties of soils were determined according to the standard methods recommended by the Ministry of Ecology and Environment of the People’s Republic of China [19]. The determination of all soil samples was carried out by random sampling, and each sample was repeated three times. The pH values were determined at a 1:2.5 ratio (sample–0.01 M CaCl_2_ solution, *w*/*v*) by a calibrated pH meter (SevenExcellence, MettlerToledo, Shanghai, China). Soil salinity was measured in a deionized water suspension (1:5, sample–water, *w*/*v*) by a calibrated electronic-conductivity (EC) meter (SevenExcellence, MettlerToledo, Shangai, China). The loss on ignition method, measuring the percentage of weight loss, was employed to quantify the organic matter (OM) content in soil samples [20]. The available nitrogen (AN) was determined by the Kjeldahl nitrogen determination method, and the available phosphorus (AP) was determined by the Olsen-P method, referring to Wang et al. [21]. The soil catalase (CAT) was extracted from prepared soil samples by enzyme kits (CAT 100, Sigma-Aldrich, Schnelldorf, Germany), the enzyme activities of which were obtained using a microplate spectrophotometer (iMark, BIO-RAD, Hercules, CA, USA) [22]. Ultrasonic-assisted solvent extraction and a GC-FID system were applied to determine the contents of total petroleum hydrocarbons (TPHs) [23]. A system of high-performance liquid chromatography (HPLC) equipped with a fluorescence detector (Agilent, Santa Clara, CA, USA) was utilized to analyze PAH concentrations, and the specific operation method can be found by referring to Li et al. [24]. Following the method reported by Sun et al. [25], the BaP-based total toxic equivalent quantity (TEQ_BaP_) was calculated.

### 2.3. DNA Extraction, Amplification, and Sequencing

Using a Soil DNA Kit (OMEGA biotech, Norcross, GA, USA), total bacterial genomic DNA samples were extracted, following the manufacturer’s instructions. After extraction, the genomic DNA of samples was stored at −20 °C prior to further analysis.

PCR amplification of the bacterial 16S rRNA genes’ V3-V4 region was performed using the forward primer 338F (5′-ACTCCTACGGGAGGCAGCA-3′) and the reverse primer 806R (5′-GGACTACHVGGGTWTCTAAT-3′). Primers to amplify C12O genes were designed by Shen et al., consisting of 32 mer forward primer C12OF (5′-GGC ACC AAG GGC AGC ATC GAG GGC CCG TAC TAC-3′) and 33 mer reverse primer (5′-CAG GTG CAG GTG CGC GGG CCG CCA CGG ATG GCC-3′) [26]. The thermal cycling consisted of initial denaturation at 98 °C for 2 min, followed by 25 cycles consisting of denaturation at 98 °C for 15 s, annealing at 55 °C for 30 s, and extension at 72 °C for 30 s, with a final extension of 5 min at 72 °C. After the purification and individual quantification step, amplicons were pooled in equal amounts, and paired-end 2 × 300 bp sequencing was performed using the Illumina MiSeq platform with MiSeq Reagent Kit v3 at Shanghai Personal Biotechnology Co., Ltd. (Shanghai, China).

Sequences sharing ≥97% similarity were clustered into identical operational taxonomic units (OTUs). Taxonomic classification and alpha diversity metrics were subsequently processed through the Genes Cloud Platform (www.genescloud.cn (accessed on 20 March 2024)). The co-occurrence networks among total bacteria and functional genes were analyzed by the software R 3.6.1 and visualized by the software Gephi 0.10.1 [27]. Based the relative abundance at the genus level in all samples, data with a cumulative abundance below 0.5% or detected in fewer than 3 samples were filtered out. According to Barberan et al. [28], genera have a strong and significant correlation when their Spearman’s correlation coefficient exceeds 0.6, coupled with a *p*-value less than 0.01. For comparison with the real network, Erdős-Rényi model-based random networks were also generated, with equivalent numbers of nodes and edges [29].

### 2.4. Real-Time Quantitative PCR

The abundances of total bacteria (16S rRNA gene) and PAH degradation bacteria carrying genes of PAH-RHDα and C12O were quantified by the MA-6000 Real-Time PCR system (Molarray, Suzhou, China). The primer sets designed by Ding and his partners were used, targeting PAH-RHDα genes, consisting of the 20-mer degenerated forward primer PAH-RHDα-396F (5′-ATT GCG CTT AYC AYG GBT GG-3′) and the 21-mer degenerated reverse primer PAH-RHDα-696R (5′-ATA GGT GTC TCC AAC RAA RTT-3′) [30]. The qPCR reaction mixture comprised 8 µL of DNA template, 8 µL of each 0.4 µL primer (Personalbio, Shanghai, China), and 10 µL of SYBR Green Master Mix (Vazyme, Nanjing, China). The thermocycling steps for the qPCR conditions were as follows: 95 °C for initial 5 min, followed by 40 cycles of 15 s at 95 °C, and then 30 s at 60 °C. A melting curve was analyzed to check the specificity and visualized in agarose (1%) gels. The absolute abundances of the 16S rRNA gene and PAH degradation gene were calculated based on their respective standard curves.

### 2.5. Statistical Analysis

All statistical analysis was performed using SPSS 17.0 (IBM, Armonk, NY, USA). Analysis of variance was used to assess significant differences among sampling sites at *p* < 0.05. Histograms were plotted via OriginPro 2018 software (OriginLab, Northampton, Ma, USA). Redundancy analysis (RDA) was performed using Canoco 5.0 software. Spearman correlational analysis and Mantel test analysis were both carried out in the R environment.

## 3. Results

### 3.1. Soil Properties and PAH Contents

Table 1 shows the soil properties in different sampling sites with six physicochemical factors. The results show that the pH of the soil in the study area ranges from 7.71 ± 0.03 to 7.96 ± 0.25. This indicates a slightly alkaline condition. The EC values range from 1.24 ± 1.05 ms·cm^−1^ to 7.23 ± 2.32 ms·cm^−1^, which indicates that the soil in the study area has a relatively high salinity and a high degree of salinization. Furthermore, the EC value decreases in a gradient from the coastal area to the inland area, with a significant difference (*p* < 0.05), indicating that the distance from the coast has a significant effect on the distribution of soil salinity. The range of OM content is from 19.87 ± 26.10 g·kg^1^ to 169.32 ± 68.35 g·kg^1^. There is no obvious pattern between bare soil and vegetated soil at the same site. The OM content in inland soil is significantly higher than that in coastal areas. CAT values range from 2.39 ± 0.61 to 4.81 ± 1.16 µmol·h^−1^·g^1^. The ranges of AN and AP are 39.51 ± 16.86 to 50.37 ± 6.26 g·kg^−1^ and 7.79 ± 4.45 to 4.05 ± 0.94 g·kg^−1^, respectively. These results show that the characteristics of alkalinity, salinity, and a low nutrient content are common to the soils of the three regions. Among them, the coastal GCB group has the highest salinity and the poorest nutrient status. When comparing the bare land and the vegetated soil, it can be found that the EC values of the samples in the vegetated area are all relatively low, while the CAT values are relatively high. This indicates that the vegetated soil in this study has a lower salinity and a higher nutrient status.

The concentrations of TPHs ranged from 5.69 ± 7.82 to 0.94 ± 0.19 mg·g^1^. Although the TPH concentration of the fourth quartile was 3.11–15.02 mg·g^−1^, only three samples exceeded the upper criteria (4.50 mg·g^−1^) required by the Chinese national standard for the second type of construction land. It is worth noting that the data out of the limit were all from bare soils. In the same sampling region, TPH concentrations in vegetated soils were all lower than those in bare soils.

PAHs were detected in all the collected soil samples. For the total PAHs (TPAHs), the concentrations ranged from 2.30 ± 0.64 to 0.79 ± 0.20 mg·kg^1^. According to the classification system recommended by Maliszewska-Kordybach [31], about 8% of soil samples in the present study were slightly polluted by PAHs, whereas 70% were heavily polluted (higher than 1.00 mg·kg^−1^). Nearly all samples from bare soils were heavily polluted by 16 PAHs. Of samples with a slight PAH contamination, about 94% were from vegetated soils. Almost all PAH concentrations in vegetated soils were found to be lower than those in bare soils, except one sample from GB. When comparing the composition of PAHs between vegetated soil and bare soil, we found no significant difference in the percentage of low-molecular-weight PAHs (LMW PAHs) among TPAHs, while HMW contributed a high percentage of TPAHs. The TEQ_BaP_ values of the 16 PAHs were also calculated to assess the toxicity of PAHs in soils. The results showed that TEQ_BaP_ values of vegetated soils were significantly lower than those of bare soils from the same sampling site. Therefore, we hypothesized that in aging, oil-contaminated soils, HMW PAHs accumulate and are the main source of toxicity. Vegetative cover may, to some extent, accelerate the removal of PAHs from oil-contaminated soils. 

### 3.2. Bacterial Community Structure and Diversity

**Figure 2:**
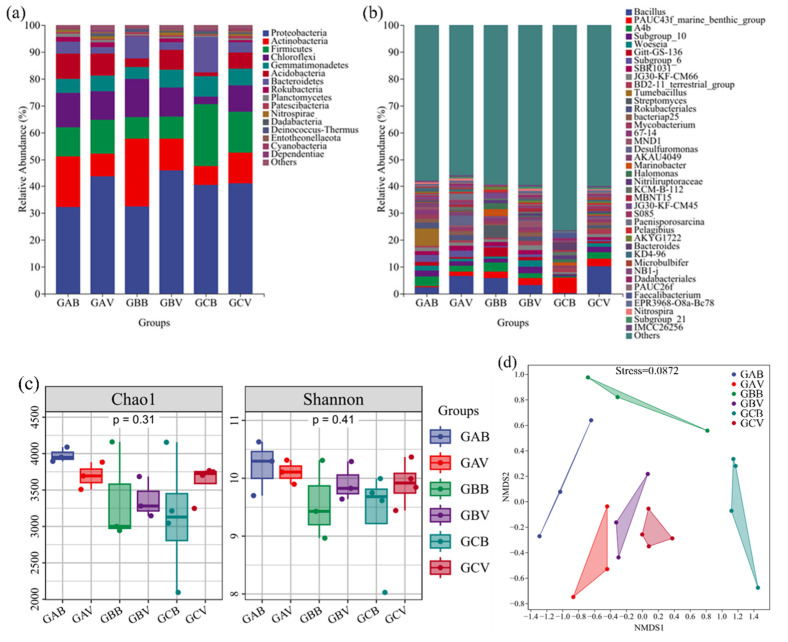
The relative abundance of the bacterial community at the phyla level (**a**). Alpha diversity of the soil bacterial communities (**b**). Diversity indexes of total bacterial community (**c**). NMDs of bacterial community (**d**).

In this study, 1,209,498 bacterial sequences were detected, ranging from 51,389 to 91,851 for each soil sample. The obtained sequences were divided into 45 phyla, 414 classes, 259 orders, 752 families, and 2690 genera, and the specific results are shown in Figure 2. Figure 2a shows that at the phylum level, the bacterial communities were composed of Proteobacteria (ranging from 22.51 to 58.12%), Firmicutes (1.21–39.38%), Actinobacteria (4.24–37.35%), Chloroflexi (1.52–21.33%), Gemmatimonadetes (3.32–10.83%), and so on. As the dominant phylum, Proteobateria showed a higher relative abundance in vegetated soils than that in bare soils in the same sampling region. As the soil salinity increased, the relative abundance of Chloroflexi and Acidobacteria showed a decreasing trend. Notably, the GCB samples exhibiting the highest soil salinity levels, and they demonstrated microbial community structures that diverged significantly from the other samples across all taxonomic levels. In samples from GCB, the relative abundance levels of Firmicutes and Bacteroidetes were higher than those in the other samples. At the class level, Deltaproteobacteria affiliated to Proteobacteria showed considerably higher abundances in vegetated soils compared to bare soils. However, in regions with higher salinity, Gammaproteobacteria affiliated to Preoteobacteria showed less abundance in vegetated soils (GCV) than in bare soils (GCB). At the genus level (Figure 2b), Bacillus (Firmicutes, Bacilli) was one of the major bacterial groups in both bare soils (accounting for 0.11–14.93%) and vegetated soils (1.34–25.31%). PAUC43f_marine_benthic_group affiliated to Gemmatimonadetes were the dominant bacteria in samples from GCB, with abundance levels that ranged from 2.35% to 9.54%, much higher than those in other soils.

The diversity results are shown in Figure 2c, based on the Chao1 and Shannon index; soils from GAB, which had a high PAH concentration and low salinity, showed the highest alpha diversity among all regions. When comparing soils of different types from the same region, vegetated soils showed a higher bacterial diversity and richness than bare soils, except for GAB. In particular, in the region with high soil salinity, values of Chao1 in vegetated soils (GCV) were significantly higher than those in bare soils (GCB) (*p* < 0.05).

To determine the effects of vegetation and soil salinity on soil bacterial communities, the NMDS analysis was applied based on the Bray–Curtis distance matrix method (Figure 2d). Among the sampling regions with different soil salinity and vegetation conditions, the structure of bacterial communities shifted considerably. Although soils from different sampling regions showed a gradient of soil salinity, samples of vegetated soils (GAV, GBV, and GCV) were clustered together and separated from samples of bare soils. When comparing regions, GAV and GAB were the most similar, with the lowest soil salinity among study regions.

### 3.3. Microbial Functional Profiling

**Figure 3:**
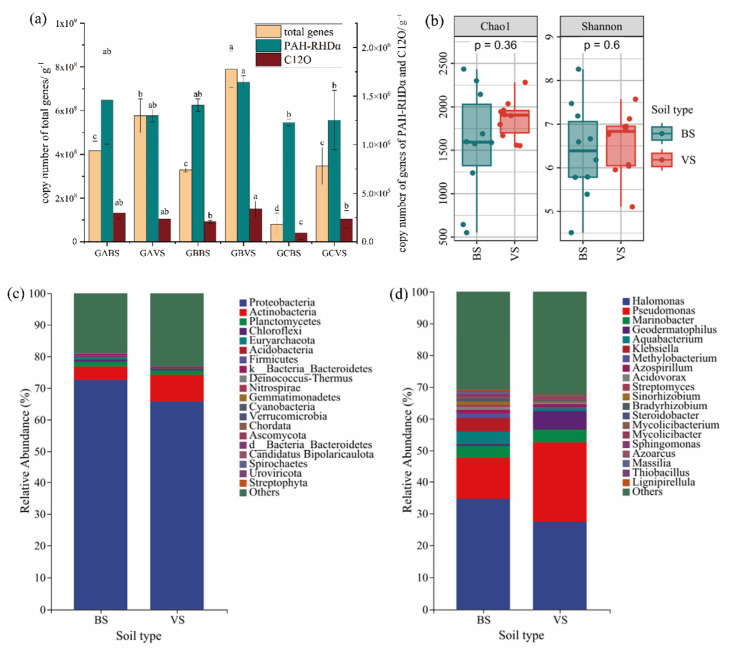
Functional bacterial communities carrying PAH-RHDα and C12O genes. (**a**) Copy numbers of total bacteria represented by 16S rRNA genes and functional bacteria represented by genes of PAH-RHDα and C12O. (**b**) Alpha diversity index of bacteria carrying C12O genes from vegetated and bare soils. (**c**) Community structure of bacteria carrying C12O genes at genus level. (**d**) Community structure of bacteria carrying C12O genes at phylum level. Error lines showed standard deviations of three replicates of the same sample. Letters above error lines indicated significant difference between sampling sites (*p* < 0.05).

The functional genes, encoding for relative enzymes, are usually used as critical indicators of microorganisms with specific functions, such as degradation and transformation of organic pollutants in soils [32]. In this study, PAH-RHDα genes encoding for ring-hydroxylating dioxygenase and C12O genes encoding for catechol 1, 2 dioxygenase, both of which represent the key steps during the biodegradation of PAHs, were quantified to evaluate the abundance of potential PAH degraders carrying these genes. The copy numbers of 16S rRNA genes representing the total bacteria were also quantified as a comparison (Figure 3a). The results showed that the copy number of total bacteria ranged from 7.19 × 10^7^ copies per gram of dry soil to 8.44 × 10^8^ copies g^−1^ with a mean value of 4.02 × 10^8^ copies g^−1^, while PAH-RHDα genes ranged from 1.04 × 10^6^ copies g^−1^ to 1.96 × 10^6^ copies g^−1^ with a mean of 1.37 × 10^6^ copies g^−1^. The copy number of C12O genes was much lower, which ranged from 7.52 × 10^4^ copies g^−1^ to 3.93 × 10^5^ copies g^−1^ with a mean of 2.26 × 10^5^ copies g^−1^. The abundance of PAH-RHDα and C12O genes showed a similar variation tendency among the three regions with a gradient of soil salinity, except for the GBV group. In bare soils, there was a significant decrease in the number of copies of both functional genes as the salt content increased (*p* < 0.05). In particular, the C12O gene was limited more clearly by soil stress than the PAH-RHDα gene. However, this variation trend of gene copy numbers was different in vegetated soils. The abundance of the two functional genes was even higher in the mid-salinity region (GBV) than in the low-salinity region (GAV).

In the same region, bacterial community and functional genes exhibited higher abundances in vegetated soils than those in bare soils. In the GA region, which had high PAH contamination, the abundances of the two functional genes were higher in bare soils (GAB) than those in vegetated soils (GAV). However, in the GB region with a higher soil salinity, the abundances of the two functional genes in vegetated soils were generally higher than those in other regions. Meanwhile, the quantities of the two functional genes were exceptions in the region GA, which showed the highest PAH concentrations. Although soils in GCV expressed high salinity, the abundances of functional genes showed no significant difference compared to those in bare soils from regions with low salinity. Compared with the C12O gene, the abundance of the PAH-RHDα gene was not significantly affected by the soil type.

As the downstream genes of PAH metabolism, C12O genes could represent the potential of deep metabolism of PAHs by bacteria in soils. In order to discuss the different occurrence characteristics of potential PAH degraders between bare and vegetated soils, the composition of bacteria carrying C12O genes were analyzed. The obtained functional sequences were divided into 20 phyla, 34 classes, 76 orders, 127 families, and 232 genera. At the phylum level (Figure 3c), the functional bacterial communities in bare and vegetated soils were both dominated by Proteobacteria (ranging from 40.48 to 94.85%), Actinobacteria (0.16–35.53%), and Planctomycetes (0.07–5.15%). As the dominant phyla, Proteobateria showed a higher relative abundance in bare soils than in vegetated soils. Meanwhile, the relative abundance of phylum Actinobacteria was higher in vegetated soils. At the genus level (Figure 3d), *Halomonas*, *Pseudomonas*, *Marinobacter*, *Geodermatophilus*, *Aquabacerium*, *Klebsiella*, and *Methylobacerium* predominated. Under conditions of relatively high salt stress in bare soils, the functional bacterial community exhibited a higher abundance of Halomonas, a genus recognized for its halophilic properties. In vegetated soils, the abundance of Pseudomonas, a genus renowned for its efficiency in degrading PAHs, was significantly higher.

Alpha diversities of bacteria carrying C12O genes from vegetated and bare soils were investigated via the Chao1 and Shannon index. The results in Figure 3b show that the diversities of functional bacteria between the two soil types had no significant difference. However, the median values of the Chao1 and Shannon index from vegetated soils were both higher than those from bare soils. The diversity index of functional bacteria in bare soils varied significantly across different samples, whereas that of vegetated soils exhibited a more homogeneous distribution.

### 3.4. Correlations Between Environmental Factors and Bacterial Communities

**Figure 4:**
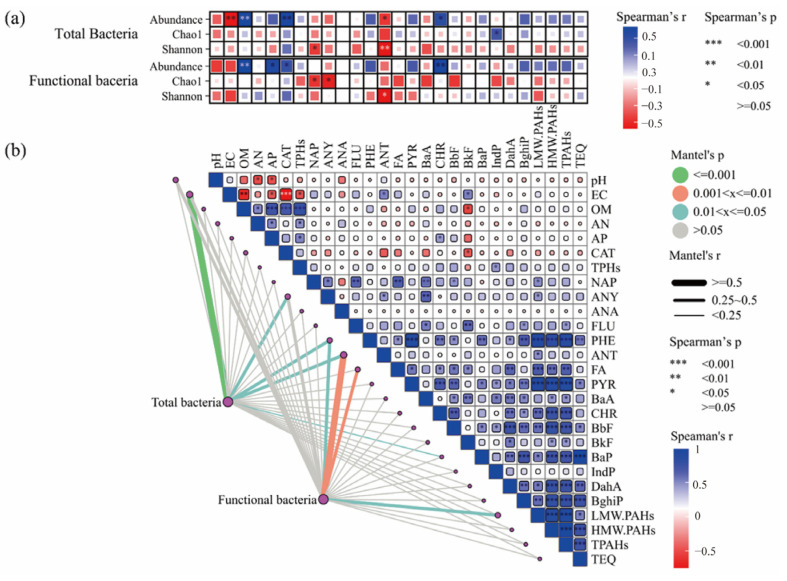
Correlations between environmental factors and the bacterial communities. (**a**) Spearman correlations between environmental factors and the abundance and diversity index of the total and functional bacteria. (**b**) The Spearman correlations between soil properties and contaminants are shown in the upper right corner, and the Mantel correlations (based on Bray–Curtis dissimilarity) between the total and functional bacterial communities and the environmental factors are shown in the lower left corner.

**Figure 5:**
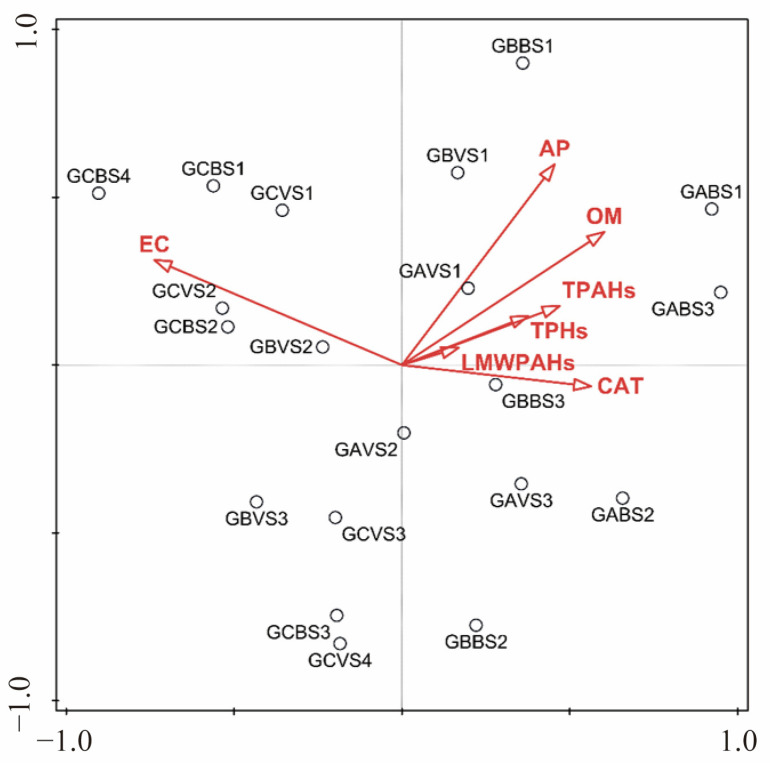
The RDA plot of relationships between soil properties and the diversity index of the total bacterial community and functional genes.

Correlations between soil physicochemical properties, contaminants, biological factors, and communities of bacteria carrying functional genes were explored. As shown in Figure 4a, there was a consistent negative correlation between soil pH and EC (soil salinity) and microbial abundance and diversity for both total bacteria and functional bacteria (*r* < 0.5). Pearson correlation analysis revealed that the CAT activity was significantly correlated with ANT and BkF (*p* < 0.05) (Figure 4b). Regarding bacterial communities, soil physicochemical properties such as OM, AP, and CAT had significant positive relationships (*p* < 0.05) with the abundance and diversity of both total and functional bacteria. Contaminants including PHE of LMW PAHs and including PYR, BaA, CHR, BaP, BbF, IndP, DahA, and BghiP of HMW PAHs could positively affect the functional bacterial communities, while FLU and ANT had a significant negative effect on both the total and functional bacteria. Through the Mantel test, the soil EC, ANT, and BkF were revealed to make significant contributions to both the total and functional bacterial communities (*p* < 0.05). Furthermore, the RDA confirmed that the environmental factors (EC, OM, CAT, and AP), when compared with the soil contaminants (ranked by explains, %), were significantly more responsible for the abundance, richness, and diversity of the bacterial community (Figure 5, Table S2).

### 3.5. Bacterial Interaction Patterns

**Figure 6:**
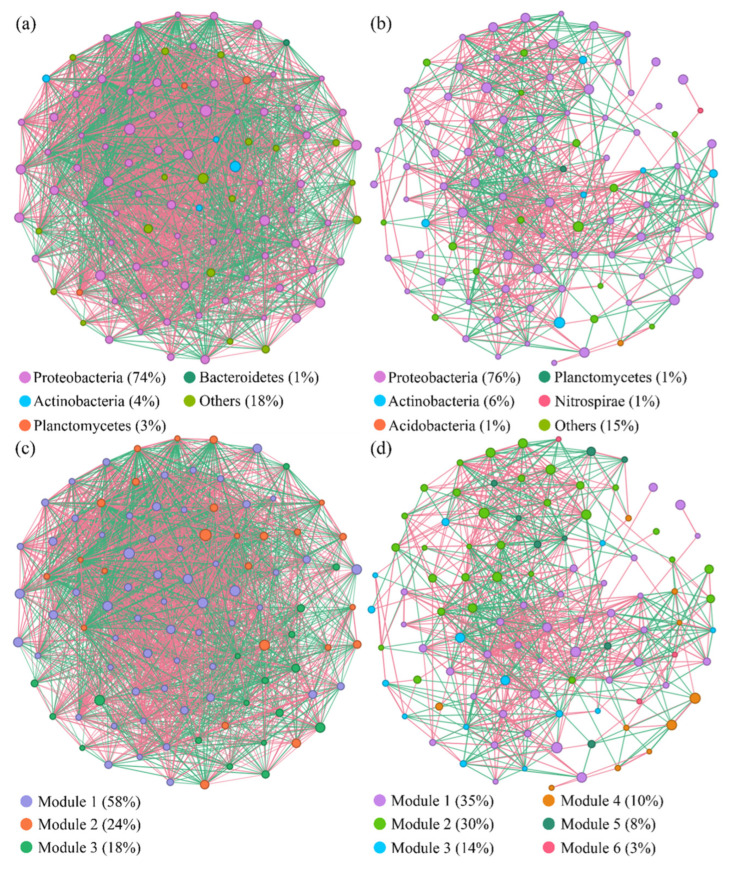
Co-occurrence networks of bacterial communities carrying C12O genes in the vegetated (**a**,**c**) and bare (**b**,**d**) soils. Nodes in the diagram represent different phyla (**a**,**b**) and different modularity classes (**c**,**d**). The red line represents a positive correlation between bacteria, while the green line represents a negative correlation.

Network analysis was applied to explore the interaction patterns among functional bacterial communities carrying C12O genes in both vegetated and bare soils. Figure 6 shows the co-occurrence network of functional bacterial communities constructed as per the dominant species of the top 100 nodes based on average abundance. The topological parameters of the network are described in Table S3. In the network of vegetated soils, 291 nodes and 21,975 edges were obtained, which were both significantly higher than those in the bare soils (220 nodes and 3088 edges). The proportion of positive correlations of all links in the network of vegetated soils (56.31%) was similar to that of bare soils (56.06%).

The modularity values of vegetated and bare soil were 0.441 and 0.450, respectively, which suggested that the real networks had modular structures (modularity values >0.4) (Table S3). In addition, compared with identically sized random networks, based on the Erdős-Rényi model, the modularity, clustering coefficient, average degree, and average path length of the real networks were all greater, suggesting that the real networks of the two communities from two types of soil both had a “small world” topology and modular structure [33]. After the node distribution was modularized, three major modules in the vegetated soils and six major modules in the bare soils were obtained (Figure 6).

The nodes in the co-occurrence network of vegetated soils mainly belong to Proteobacteria (74%), Actinobacteria (4%), Planctomycetes (3%), and Bacterodetes (1%) (Figure 6). Meanwhile, the nodes of bare soils mainly belong to Proteobacteria (76%), Actinobacteria (6%), Acidobacteria (1%), Planctomycetes (1%), and Nitrospirae (3.8%). Based on the betweenness centrality, genera of Halomonas, Pseudomonas, and Geodermatophilus were identified as the potential keystone taxa of PAH-degrading bacteria in vegetated soils, except for uncultured and unidentified bacteria. Meanwhile, in the bare soils, the top genera were Halomonas, Pseudomonas, and Methylobacterium (Table S4).

## 4. Discussion

### 4.1. Natural Revegetation Affected the Soil Properties and PAH Contents

Oil alkalinization, compaction, nutrient deficiency, and low vegetation coverage are the common natural conditions in the YRD, which lead a fragile ecological environment for indigenous organisms in local soils [34,35]. After long-term adaptation and development, halophytes have acclimated to the infertile and high-salinity soil environment and become the primary vegetation of the local ecosystem. Root systems of halophytes in saline soils can regulate the salt content, thereby influencing both soil physicochemical properties and the microbial community structure, fostering a beneficial microenvironment for plant growth and development [36,37].

Although the three sampling regions showed a gradient of soil salinities, it was revealed that they had a similar environmental status, affected significantly by vegetation in each group. This phenomenon was particularly pronounced in the GC group, which showed the highest soil salinities. Salinity levels in the vegetated soils from the GC region were much lower than those in bare soils, which could be owing to halophytes such as Suaeda. The plant species of Suaeda salsa were reported to have the ability to absorb salts from the soil and store them in their fleshy stems and leaves [38]. In addition, vegetated soils also showed higher nutrient levels, such as OM, CAT, AP, and AN, which could be mainly attributed to the root systems and litters of these salt-tolerant plants [39,40].

Since the soils were collected at closed, abandoned oil well sites, the concentrations of TPAHs obtained in this study exhibited a higher range and mean value compared to data reported previously in the YRD, for example, the results from Hongming Yuan et al. [41] (27–753 mg·kg^−1^ with a mean of 118 ± 132 mg·kg^−1^) and Zijiao Yuan [42] (79.2–311 mg·kg^−1^ with a mean of 119 mg·kg^−1^). In the present study area, accidents of oil well blowout, pipeline leakage, and improper operation leakage could be responsible for the elevated levels of PAH contamination in soils [43]. However, after natural revegetation in these soils, the concentrations of both TPAHs and LMW PAHs were significantly lower than those in bare soils from the same sampling regions. Consistent with this, the toxic risk of PAHs, indicated by the index of TEQ_BaP_, also exhibited lower levels in vegetated soils. These results demonstrated that vegetation restorations at aged, PAH-contaminated sites have a positive effect in reducing the concentrations and toxicities of PAHs [44]. Moreover, this effect is primarily targeted at the LMWPAHs. Previous studies have demonstrated that plants can absorb and degrade low-molecular-weight organic compounds, including LMW PAHs [45,46]. Therefore, this study verified that the planting of vegetation can reduce soil salinity to a certain extent and promote the attenuation capacity of polycyclic aromatic hydrocarbons in the soil, especially for the occurrence of HMW PAHs in the soil.

### 4.2. Natural Revegetation Affected the Components and Diversity of Bacterial Communities

In this study, the bacterial communities in soils from aged, PAH-contaminated sites are simultaneously exposed to dual pressure from soil salinization and PAH contamination. The extreme environmental conditions filter out bacteria with low salt tolerance and restrain the abundance of those adapted to high salinity. However, under high salinity stress, the total bacterial communities in vegetated soils showed higher abundance and diversity than bare soils. Many research studies have indicated that plants can help soil microorganisms resist the adverse effects of environmental factors [47,48]. Pan et al. [49] reported that by improving soil physicochemical properties, such as salinity, pH, and soil porosity, plants provide a more suitable habitat for existing microorganisms, thereby increasing the abundance and diversity of soil microorganisms. Through secreting root exudates, such as small-molecule acids and sugars, plants play a screening role in the growth and reproduction of soil microorganisms, thus altering the microbial community structure [50].

Through correlation analysis, environmental factors were revealed to contribute more significantly to the structure of bacterial communities than pollutants of PAHs and TPHs. Bacterial communities in the vegetated soils showed more cooperative behaviors than those in the bare soils under long-term exposure to organic contamination and soil salinization. This phenomenon could be attributed to the mitigating effect of vegetation on microorganisms under the stress of soil contamination. In addition, this effect was probably mediated through alterations in soil physicochemical properties by local vegetation. Former research demonstrated that vegetation-induced modulation of soil properties and microbial consortia could transcend the rhizosphere boundary into the bulk soil environment [51].

The roots of plants significantly impact the formation of essential bacterial species within the soil microbial community. In contrast to bare soils, the co-occurrence network of the vegetated soil bacterial community exhibited enhanced connectivity and greater modularity, which could be regarded as a heightened capacity to withstand and recover from external environmental disturbances [52]. In vegetated soils, numerous low-abundance bacteria, such as *Entotheonellaeota*, *Dadabacteria*, and *Latesbacteria*, demonstrate high network connectivity. These microorganisms play crucial ecological roles in maintaining the diversity and structural stability of the rhizosphere bacterial community, especially when facing environmental risks, such as soil salinization and contaminations [53]. Furthermore, a comparative analysis of vegetated and bare soils revealed that the majority of significantly differing bacteria belong to the Proteobacteria phylum. These findings offer valuable insights for the future selection of multi-functional rhizobacteria.

Therefore, it can be concluded that soil salinization and polycyclic aromatic hydrocarbon pollution have seriously affected the life activities of microorganisms in aged, petroleum-polluted soil, leading to a decrease in the bioavailability of the contaminated soil. However, the cultivation of plants can to a certain extent buffer the toxic effects of salinity and PAHs on microorganisms, change the physical and chemical properties of the soil, improve the symbiotic network connection between microorganisms and the living environment of plants, and enhance the defense ability against external risks. For coastal cities contaminated by PAHs, increasing the coverage rate of salt-tolerant related debris might be a practical method for the plasmid soil environment.

### 4.3. Natural Revegetation Stimulated the Biodegradation Potential of PAHs

Functioning as vital decomposers within ecosystems, bacterial communities play a crucial role in driving biogeochemical processes and regulating energy transfer [54]. In vegetated soils, the taxonomic and functional diversity of these bacteria can be affected by the accumulation of soil nutrients derived from stimulated nutrient cycling potential [55].

By counting gene copies of bacteria carrying C12O and PAH-RHDα genes from both vegetated and bare soils, the biodegrading potential of PAHs was revealed to be significantly affected by vegetation conditions under salt stress. Consistent with the report of Gao et al. [56], the abundance of functional bacteria was dominated by soil properties and vegetation conditions. In high-salinity soils of this study, the abundance of bacteria carrying C12O genes was much higher in vegetated soils, which meant that the PAH-degrading potential was raised by revegetation. When salt stress severely inhibited the biomass in bare soils, the functional bacteria in vegetated soil showed an equal abundance to those without salt stress. This result suggested an alleviation for functional bacteria communities of the negative effects of soil salinization. Wang et al. [57] reported increased catabolic activities of soil bacteria caused by the succession of halophytic vegetation, which led them to make a similar conclusion about the improvement of bacterial function by vegetation under adverse conditions.

When comparing the abundances of the two types of functional genes, C12O exhibited a more sensitive response to salt stress and was also more significantly influenced by vegetation growth. When analyzing the structure and diversity of bacteria carrying C12O genes in bare and vegetated soils, we found that the dominant phyla and genera of functional bacteria between the two soil types showed no significant difference, but their relative abundances were altered. Based on the correlation analysis results, a significant relationship between the characteristics of bacterial communities and soil physicochemical properties was revealed. Similar to the response of the total bacterial community discussed in this study, this effect of vegetation on functional bacterial communities could also be attributed to its amelioration of soil properties [58]. Likewise, Guo et al. [59] found that revegetation could not only modify the abundance of functional groups but also adjust microbial-mediated biomass decomposition processes.

In addition, the co-occurrence network analysis revealed the positive influences of vegetation in terms of an improvement of relations, connectivity, and keystone species of PAH-degrading bacteria in vegetated soils. These shifts of bacterial communities were probably the reason for the lower pollutant concentration and toxicity (TEQ_BaP_) in vegetated soils. Previous research demonstrated that vegetation could mitigate the inhibitory effects of soil salinity stress on microbial viability and enhance the dissipation efficiency of pollutants [60]. Research has revealed that plants strategically recruit functional microbial consortia to mitigate abiotic stresses, including soil contamination and soil salinity [55,61]. In essence, vegetation exert a dual modulation on soil condition amelioration and functional microbiome reorganization, establishing a synergistic interplay among soil, plants, and microorganisms.

## 5. Conclusions

In this paper, when discussing the characteristics of habitat conditions, the total bacterial community, and PAH-degrading bacteria in PAH-contaminated soils after decades of succession, natural revegetation was revealed to have an ability to improve the soil ecosystem. Compared to bare soils, vegetation communities were suggested to alter the taxonomic structure of soil bacterial communities, improve the bacterial diversity, and enhance the PAH-degrading potential under different salinity levels. Vegetation restoration was found to mediate the abiotic pressure and improve the network connectivity of bacterial communities, which exert a profound influence on ecological processes. The results provide a comprehensive understanding of the influences of natural revegetation restoration on the taxonomic characteristics and functional capability of bacterial communities in aged, contaminated regions.

## Figures and Tables

**Table 1:** Physicochemical and biological parameters in soils from each sampling site.

Item	GAB	GAV	GBB	GBV	GCB	GCV
pH	7.71 ± 0.03 a	7.81 ± 0.08 a	7.81 ± 0.16 a	7.74 ± 0.09 a	7.96 ± 0.25 a	7.85 ± 0.22 a
EC (ms·cm^−1^)	1.25 ± 0.70 b	1.24 ± 1.05 b	2.06 ± 0.37 b	1.98 ± 1.17 b	7.23 ± 2.32 a	2.96 ± 1.84 b
OM (g·kg^−1^)	132.65 ± 77.84 ab	138.00 ± 24.66 ab	169.32 ± 68.35 a	102.27 ± 69.95 ab	19.87 ± 26.10 b	36.95 ± 11.01 b
CAT (μmol·h^−1^·g^−1^)	4.07 ± 0.46 ab	4.81 ± 1.16 a	3.52 ± 0.17 b	3.49 ± 1.09 b	2.39 ± 0.61 b	3.42 ± 0.36 b
AN (g·kg^−1^)	50.37 ± 6.26 a	49.21 ± 6.10 a	47.48 ± 17.57 a	42.71 ± 18.21 a	41.25 ± 6.08 a	39.51 ± 16.86 a
AP (g·kg^−1^)	7.19 ± 1.96 c	7.20 ± 1.17 c	7.79 ± 4.45 c	5.86 ± 2.88 c	4.05 ± 0.94 a	5.60 ± 2.40 b
TPHs (g·kg^−1^)	5.69 ± 7.82 a	2.43 ± 0.89 a	5.88 ± 7.92 a	2.41 ± 1.23 a	1.74 ± 2.07 a	0.94 ± 0.19 a
TPAHs (mg·kg^−1^)	2.30 ± 0.64 a	1.13 ± 0.21 b	2.04 ± 0.56 ab	0.90 ± 0.38 b	1.77 ± 0.43 a	0.79 ± 0.20 b
LMW PAHs (mg·kg^−1^)	0.46 ± 0.05 ab	0.38 ± 0.11 b	0.63 ± 0.07 a	0.27 ± 0.13 b	0.62 ± 0.25 a	0.21 ± 0.11 b
HMW PAHs (mg·kg^−1^)	1.84 ± 0.63 a	0.76 ± 0.10 a	1.07 ± 0.04 a	0.63 ± 0.26 a	1.16 ± 0.19 a	0.58 ± 0.10 a
TEQ_BaP_ (mg·kg^−1^)	0.39 ± 0.13 a	0.03 ± 0.002 b	0.11 ± 0.06 b	0.08 ± 0.06 b	0.15 ± 0.09 b	0.06 ± 0.05 b

Lowercase letters indicate significant differences between groups (*p* < 0.05, Tukey HSD test).

## Data Availability

The original contributions presented in this study are included in the article/Supplementary Materials. Further inquiries can be directed to the corresponding author.

## Supplementary Materials

The following supporting information can be downloaded at https://www.mdpi.com/article/10.3390/microorganisms13051098/s1, Table S1: Sample information of aged PAH-polluted soils in the Shengli Oilfield; Table S2: Interpretation rate of the simple effects of each environmental variable; Table S3: Topological factors of co-occurrence networks; Table S4: Keystone species in co-occurrence network based on values of betweenness centrality.
