# Supplementary material for: Natural Revegetation Alters Habitat Conditions, Bacterial Components, and Polycyclic Aromatic Hydrocarbon (PAH)-Degrading Communities in Aged PAH-Polluted Soils
Source: Microorganisms. 2025 May 9;13(5):1098. doi: 10.3390/microorganisms13051098 (PMC12114558; doi:10.3390/microorganisms13051098)
Supplement: Supplementary file 1 [file microorganisms-13-01098-s001.zip › microorganisms-3581355-supplementary.pdf]

Supplementary Information

Natural revegetation alters habitat condition, bacterial component and PAH-degrading communities in aged PAH-polluted soils

Jinrong Huang [1], Heng Liang [2], Lilong Huang [3], Qi Li [1], Lei Ji [1], Yingna Xing [1], Chang Zhou [1], Jianing Wang [1], Xiaowen Fu [1*]

1. Shandong Province Key Laboratory of Applied Microbiology, Ecology Institute, Qilu University of Technology (Shandong Academy of Sciences), Jinan 250103, China

2. Shandong Environmental Sciences Environmental Engineering Co., LTD., Jinan 250109, China

3. School of Environmental Science and Engineering, Shandong University, Qingdao 266237, China

∗ Corresponding author: Xiaowen Fu, Qilu University of Technology (Shandong Academy of Sciences), China. E-mail: fuxw@qlu.edu.cn.

Number of pages: 4 (including cover page)

Number of tables: 4

Number of figures: 0

Table S1 Sample information of aged PAHs polluted soils in the Shengli Oilfield

Sampling region	Sample name	Type	Vegetation community	Aged time /year
GA	GAB1	Bare	—	25
	GAV1	Vegetated	*Phragmites australis, Tamarix chinensis, Chloris virgata*	
	GAB2	Bare	—	30
	GAV2	Vegetated	*Phragmites australis, Tamarix chinensis*	
	GAB3	Bare	—	20
	GAV3	Vegetated	*Phragmites australis, Tamarix chinensis*	
GB	GBB1	Bare	—	12
	GBV1	Vegetated	*Phragmites australis, Suaeda heteroptera, Tamarix chinensis*	
	GBB2	Bare	—	15
	GBV2	Vegetated	*Suaeda heteroptera, Tamarix chinensis*	
	GBB3	Bare	—	17
	GBV3	Vegetated	*Suaeda heteroptera, Tamarix chinensis*	
GC	GCB1	Bare	—	10
	GCV1	Vegetated	*Phragmites australis, Suaeda heteroptera*	
	GCB2	Bare	—	15
	GCV2	Vegetated	*Phragmites australis*	
	GCB3	Bare	—	12
	GCV3	Vegetated	*Phragmites australis*	
	GCB4	Bare	—	15
	GCV4	Vegetated	*Phragmites australis*	

Table S2 Interpretation rate of the simple effects of each environmental variable

Environment variable	Explains (%)	Pseudo-F	*p* values
EC	53.7	12.0	**0.004***
OM	36.0	6.6	**0.020***
CAT	31.3	5.5	**0.030***
TPAHs	21.8	3.5	0.084
AP	21.0	3.3	0.084
TPHs	14.0	2.1	0.148
LMWPAHs	2.8	0.4	0.562

* means significant correlation when $p<0.05$

Table S3 Topological factors of co-occurrence networks

	Average degree	Average path length	Average clustering coefficient	Graph density	Network diameter	Modularity
Vegetated soil						
Real network	168.078	1.479	0.639	0.521	2	0.441
Random network	151.531	1.479	0.521	0.521	2	0.043
Bare soil						
Real network	33.802	2.375	0.545	0.128	5	0.450
Random network	28.949	1.896	0.128	0.128	3	0.150

Table S4 Keystone species in co-occurrence network based on values of betweenness centrality

	Phylum	Class	Order	Family	Genus	Betweenness centrality
Vegetated soil	Proteobacteria	Gammaproteobacteria	Oceanospirillales	Halomonadaceae	Halomonas	166.86
	Proteobacteria	Gammaproteobacteria	Pseudomonadales	Pseudomonadaceae	Pseudomonas	160.09
	—	—	—	—	—	146.65
	Proteobacteria	Gammaproteobacteria	Oceanospirillales	Halomonadaceae	Halomonas	129.23
	Proteobacteria	Gammaproteobacteria	Oceanospirillales	Halomonadaceae	Halomonas	128.68
	Proteobacteria	Gammaproteobacteria	Oceanospirillales	Halomonadaceae	Halomonas	122.83
	Proteobacteria	Gammaproteobacteria	Oceanospirillales	Halomonadaceae	Halomonas	122.15
	Proteobacteria	Gammaproteobacteria	Pseudomonadales	Pseudomonadaceae	Pseudomonas	118.88
	Actinobacteria	Actinomycetia	Geodermatophilales	Geodermatophilaceae	Geodermatophilus	111.48
	Proteobacteria	Gammaproteobacteria	Pseudomonadales	Pseudomonadaceae	Pseudomonas	109.72
Bare soil	—	—	—	—	—	1135.31
	Proteobacteria	Gammaproteobacteria	Oceanospirillales	Halomonadaceae	Halomonas	870.60
	Proteobacteria	Gammaproteobacteria	Pseudomonadales	Pseudomonadaceae	Pseudomonas	714.90
	Proteobacteria	Gammaproteobacteria	Pseudomonadales	Pseudomonadaceae	Pseudomonas	567.87
	Proteobacteria	Gammaproteobacteria	Oceanospirillales	Halomonadaceae	Halomonas	549.55
	Proteobacteria	Gammaproteobacteria	Oceanospirillales	Halomonadaceae	Halomonas	527.04
	Proteobacteria	Gammaproteobacteria	Oceanospirillales	Halomonadaceae	Halomonas	523.30
	—	—	—	—	—	519.68
	Proteobacteria	Gammaproteobacteria	Oceanospirillales	Halomonadaceae	Halomonas	506.80
	Proteobacteria	Alphaproteobacteria	Hyphomicrobiales	Methylobacteriaceae	Methylobacterium	474.33
